# Increasing incidence of pregnancy among women receiving HIV care and treatment at a large urban facility in western Uganda

**DOI:** 10.1186/1742-4755-11-81

**Published:** 2014-12-06

**Authors:** Jane Kabami, Eleanor Turyakira, Sam Biraro, Francis Bajunirwe

**Affiliations:** Department of Community Health, Mbarara University of Science and Technology, P.O. BOX 1410, Mbarara, Uganda; Medical Research Council, Entebbe, Uganda

## Abstract

**Background:**

Antiretroviral treatment restores physical functioning and may have an impact on fertility desires. Counseling is given to HIV positive women to create awareness and to provide information on pregnancy and delivery. The purpose of this study was to determine the incidence of pregnancy and factors that predict pregnancy among women of reproductive age receiving HIV care and treatment at a large urban center in western Uganda.

**Methods:**

We conducted a retrospective cohort study using routinely collected data at the Immune Suppression (ISS) Clinic of Mbarara Regional Referral Hospital located in Mbarara District, western Uganda collected between January 2006 and June 2010. Women aged 15 to 50 years were eligible for analysis. The primary outcome was incidence of pregnancy calculated as number of pregnancies per 1000 person years (PY). Data was analyzed by calendar year and year of enrolment and used survival analysis to determine the predictors of pregnancy.

**Results:**

A total of 3144 women were included with a median follow up of 12.5 months. The overall incidence rate was 90.7 pregnancies per 1000 person years. Incidence increased from 29.8 pregnancies per 1000 PY in 2006 to 122 pregnancies per 1000 PY in 2010 (p < 0.001). Significant predictors for pregnancy were younger age (HR 10.96 95% CI 3.22-37.2), married (HR 2.09 95% CI 1.69-2.64) and single (HR 1.95 95% CI 1.34-2.84) compared to widowed or separated, primary education (HR 1.65 95% CI 1.02-2.66), not knowing the HIV status of the spouse (HR 1.46, 95%CI 1.13-1.93) compared to knowing. The use of family planning (HR 0.23 95% CI 0.18- 0.30) and an increase in CD4 count between baseline and most recent count were protective against pregnancy. ART use was not a significant predictor.

**Conclusion:**

Incidence of pregnancy among women receiving routine HIV care and treatment has increased and is almost comparable to that in the general population. Thus routine HIV care should integrate reproductive health needs for these women.

**Electronic supplementary material:**

The online version of this article (doi:10.1186/1742-4755-11-81) contains supplementary material, which is available to authorized users.

## Introduction

Antiretroviral therapy (ART) is now widely available in resource limited settings following major large scale initiatives [1–4] and the treatment significantly improves the physical functioning [5–7] and sexual activity [8] of HIV infected patients. For HIV infected women, the prospects of getting pregnant and having an HIV negative baby will be significantly improved with the increasing availability of ART, because of its association with a reduction in the risk of mother to child transmission of HIV [9–11]. This may result in an increasing trend in incidence of pregnancy among HIV infected women. However, studies on fertility and HIV in sub Saharan Africa have shown that HIV infection is associated with reduced fertility [12–14] and that ART may have a potential impact on the fertility [15].

Recent studies from sub Saharan Africa have shown that HIV positive women are less likely to report fertility intentions compared to HIV negative women [16, 17], however there were no differences observed in child bearing desire between ART treated and naïve women. Prospective studies have shown that reproductive intentions and desires differ significantly from practices with more women having babies they initially did not plan to have, especially following initiation of ART [18].

HIV infection in pregnancy is associated with a variety of complications. For instance, HIV infection is associated with adverse pregnancy outcomes and substantial maternal mortality even among women with high CD4 count [19], despite the availability of ART. Maternal mortality rates have been reported to be five times higher in HIV infected women than in uninfected women and responsible for at least 20% of all deaths, a figure that is higher than any direct obstetric cause [20]. Recent data has also shown an increased risk of female to male HIV transmission during pregnancy, suggesting pregnancy as a risk factor for transmission of HIV [21].

The potential complications and risks necessitate a clear understanding of the trends and patterns in occurrence of pregnancy among HIV infected women. Clinicians and counselors at the clinics also need to identify women that are more likely to get pregnant and target them for counseling and guidance on safe pregnancy and prevention of mother-to-child transmission of HIV. Therefore, the aim of this study was to determine the incidence rate and predictors of pregnancy among HIV positive women of reproductive age attending a large urban HIV clinic at Mbarara Regional referral Hospital in southwestern Uganda.

## Methods

We constructed a retrospective cohort using routine records collected at the Immune suppression (ISS) clinic at Mbarara Regional Referral hospital (MRRH) located in Mbarara District in southwestern Uganda. The district is predominantly rural, with large majority earning a living as subsistence farmers. The regional referral facility is also the teaching hospital for Mbarara University of Science and Technology’s medical school. Data used for this study was collected routinely from patients attending their monthly visits to collect medicine refills at the ISS clinic at Mbarara Hospital. Analysis of data for this paper was restricted to women of reproductive age (15–50 years) enrolled into HIV care or ART treatment between June 2006 and January 2010.

History taken at every routine visit includes sexual activity, determination of the date of last normal menstruation period (LNMP) and clinical examination. The primary outcome for this study was incidence of pregnancy. Pregnancy was recorded if a clinical diagnosis based on history and examination was made during the follow up visits and entered into the records. The date pregnancy occurred was calculated to be date of the last normal menstrual period. All the subsequent visits following the pregnancy diagnosis were censored in the analysis.

The independent variables considered in the analysis were age, educational status, weight, religion, monthly income, marital status, occupation, total number of children, disclosure of HIV sero-status, use of PMTCT services, HIV status of the spouse, family planning use and method, ART initiation date and duration of use, religion, WHO disease stage and CD4 cell count. Three data sets were extracted; the first set comprised of constant variables such as educational status and occupation, the second had time varying variables such as weight and the last had CD4 count measures for the follow up period. The three data sets were cleaned and merged together for final analysis.

### Data analysis

Frequency distributions were used to summarize data on the categorical variables and some variables like age, family size, and education level were grouped into categories before the frequencies were generated. Means and medians were used to summarize the continuous variables. We considered change in CD4 count as an exposure factor and calculated the absolute change in CD4 count between measures at baseline and the most recent. The incidence of pregnancy was measured as number of pregnancy events per 1000 person years (PY). We used Poisson regression to estimate the incidence rates. Survival analysis with parametric survival-time models were used to obtain crude and adjusted hazard ratios for the predictor variables with 95% confidence intervals. Women were censored after their first reported pregnancy as clients in the ART clinic. The exposure variables considered in the analysis were use of ART, marital status, disclosure, number of children and their HIV status, and use of contraception. We calculated incidence rates of pregnancy over the calendar years of follow up and tested for the trends in incidence using the chi square test for trend. All the analyses were done using Stata Version 11.

### Ethical considerations

The study was approved by the ISS clinic data sharing committee, the Faculty of Medicine Research ethics committee and the Institutional review Board of Mbarara University of Science and Technology. All the datasets extracted from the Open electronic Medical Records System (OMRS) database were stored with password access and codes were used instead of names and all the information was kept confidential.

## Results

### Baseline characteristics of participants

**Figure 1:**
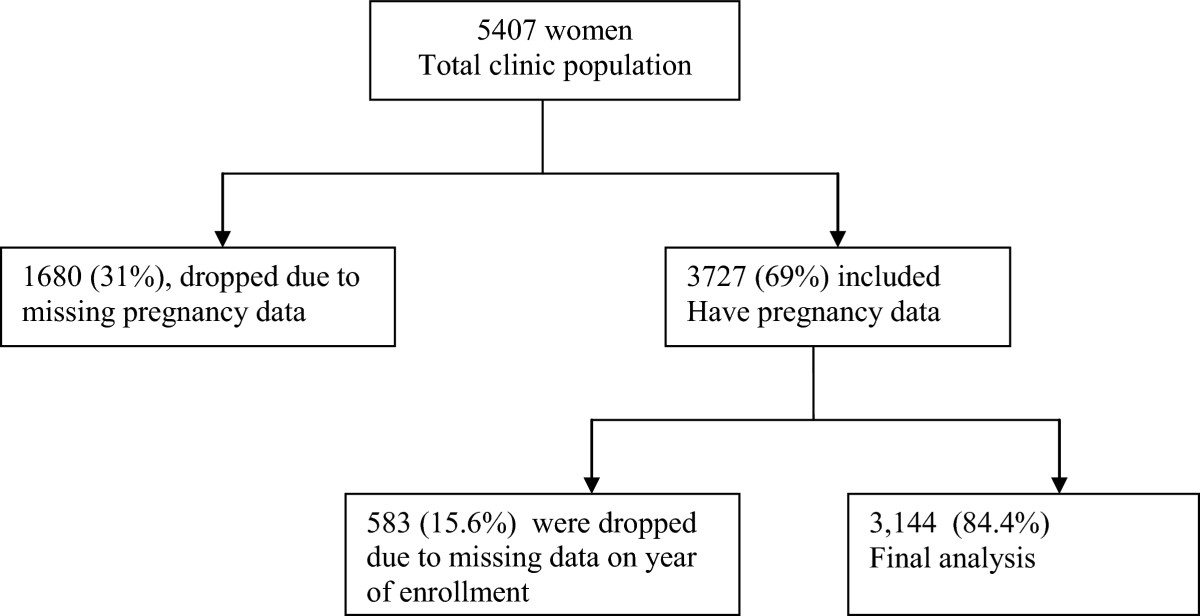
**Figure showing the flow chart of women enrolled at the clinic and those included in the final analysis.**

Data was extracted for a total of 5,407 women. Of these, 1680 women had missing data on pregnancy status and 583 were missing data on date of enrollment and were removed from the analysis. Hence 3,144 women had complete data and were considered for the final analysis. The flow diagram is shown in Figure 1. The duration of follow up ranged from 0 to 49 months, with an average follow up of 15 months (standard deviation, SD = 11, median = 12.5). The mean age was 33 years (SD = 9.3, median = 32) and more than 50% had attained primary school education (Tables 1 and 2). Peasant farmers constituted up to 37.3% of the women. Most of these women were low income earners and 56.9% earned less than 100,000 Uganda shillings (40USD) at the time of clinic enrollment. Only about one quarter (27.3%) of the women had disclosed their HIV serostatus but more than 50% of the women had their disclosure status unknown.

**Table 1:** **Baseline demographic characteristics of women of reproductive age attending Mbarara ISS clinic**

Variable	Pregnant (n = 462)	Not pregnant (n = 2,682)	Total (n = 3,144)
**Age (years)**			
39 and above	21 (4.6)	671 (25.0)	692 (22.0)
32 -38	97 (21)	703 (26.2)	800 (25.1)
25 -31	208 (45.2)	820 (30.6)	1,028 (32.7)
18 – 24	133 (28.79)	477 (17.8)	610 (19.4)
Below 18	3 (0.65)	11 (0.04)	14 (0.45)
**Education level**			
Tertiary	18 (3.9)	165 (13.2)	183 (5.8)
Secondary	84 (18.2)	440 (16.4)	524 (16.7)
Primary	282 (61.0)	1,438 (53.6)	1,720 (54.7)
None	44 (9.5)	284 (10.6)	328 (10.4)
Unknown	34 (7.4)	355 (13.2)	389 (12.4)
**Occupation**			
Formal employment	86 (18.6)	559 (20.8)	645 (20.5)
Unemployed	95 (20.6)	538 (20.1)	633 (20.1)
Peasant farmer	180 (38.9)	992 (40.0)	1,172 (37.3)
Others	59 (12.8)	314 (11.7)	373 (11.9)
Unknown	42 (9.1)	279 (10.4)	321 (10.2)
**Income**			
Above 500,000	8 (1.2)	47 (1.75)	55 (1.75)
100,000 - 500,000	20 (4.3)	221 (8.2)	241 (7.7)
Less than 100,000	263 (56.9)	1,538 (57.4)	1,801 (57.3)
Unknown	171 (37)	876 (32.6)	1,047 (33.3)
**Religion**			
Protestant	219 (47.4)	1,314 (48.9)	1,533 (48.7)
Catholic	128 (27.7)	723 (27.0)	851 (27.1)
Moslem	45 (9.7)	246 (9.2)	291 (9.3)
Seventh day/Pentecostal	5 (1.1)	31 (1.16)	36 (1.2)
Unknown	50 (10.8)	298 (11.1)	348 (11.1)
Others	15 (3.3)	70 (2.6)	85 (2.7)
**HIV status of the spouse**			
Unknown by woman	71 (15.4)	428 (15.9)	499 (15.8)
Negative	15 (3.2)	60 (2.2)	75 (2.5)
Positive	214 (46.3)	1,133 (42.8)	1,347 (42.8)
Missing data	162 (35.1)	1,061 (39.6)	1,223 (38.9)
**ART use**			
Yes	165 (35.7)	1,109 (41.3)	1,274 (40.5)
No	297 (64.3)	1,573 (58.7)	1,870 (59.5)
**Disclosure**			
No	29 (6.3)	236 (8.8)	265 (8.4)
Yes	126 (27.3)	1,075 (40.1)	1,201 (38.2)
Missing data	307 (66.4)	1,371 (51.1)	1,678 (53.4)

**Table 2:** **Table of family and clinical characteristics of women receiving HIV care and treatment at Mbarara ISS clinic**

Variable	Pregnant (n = 462)	Not pregnant (n = 2,682)	Total (n = 3,144)
**Total number of children alive**			
5 and above	274 (59..3)	1,457 (54.3)	1,731 (55.0)
2-5 children	116 (25.1)	892 (33.3)	1008 (32.1)
1 child	72 (15.6)	333 (12.4)	405 (12.9)
**Weight (kg)**			
Less than 50	71 (16.5)	672 (29.4)	743 (27.3)
51 – 60	202 (46.9)	974 (42.6)	1,176 (43.3)
61 -71	110 (25.6)	431 (18.8)	541 (19.9)
Above 71	47 (10.9)	212 (9.3)	259 (9.5)
**WHO stage**			
Stage 1	152 (32.9)	935 (34.9)	1,087 (34.6)
Stage 2	151 (32.7)	806 (30)	957 (30.4)
Stage 3	38 (8.2)	317 (11.8)	355 (11.2)
Stage 4	2 (0.4)	66 (2.5)	68 (2.2)
Missing	119 (25.6)	558 (20.8)	677 (21.5)
**Marital status**			
Married	260 (56.3)	1,034 (38.6)	1,294 (41.2)
Single	38 (8.2)	208 (7.8)	246 (7.8)
Widowed/separated	149 (32.3)	1,344 (50.1)	1,493 (47.5)
Missing data	15 (3.3)	96 (3.6)	111 (3.5)
**CD4 count difference between most recent and baseline measure**			
Less than 100	58 (12.6)	309 (11.5)	367 (11.7)
100 – 250	8 (1.7)	67 (2.5)	75 (2.4)
250 – 350	2 (0.4)	18 (0.6)	20 (0.6)
Greater than 350	2,288 (85.3)	394 (85.3)	2,682 (85.3)
**Family planning use**			
Yes	70 (15.1)	1,114 (41.5)	1,184 (37.7)
No	339 (73.4)	1,394 (52)	1,733 (55.1)
Missing	53 (11.5)	174 (6.5)	227 (7.2)

### Incidence of pregnancy

**Figure 2:**
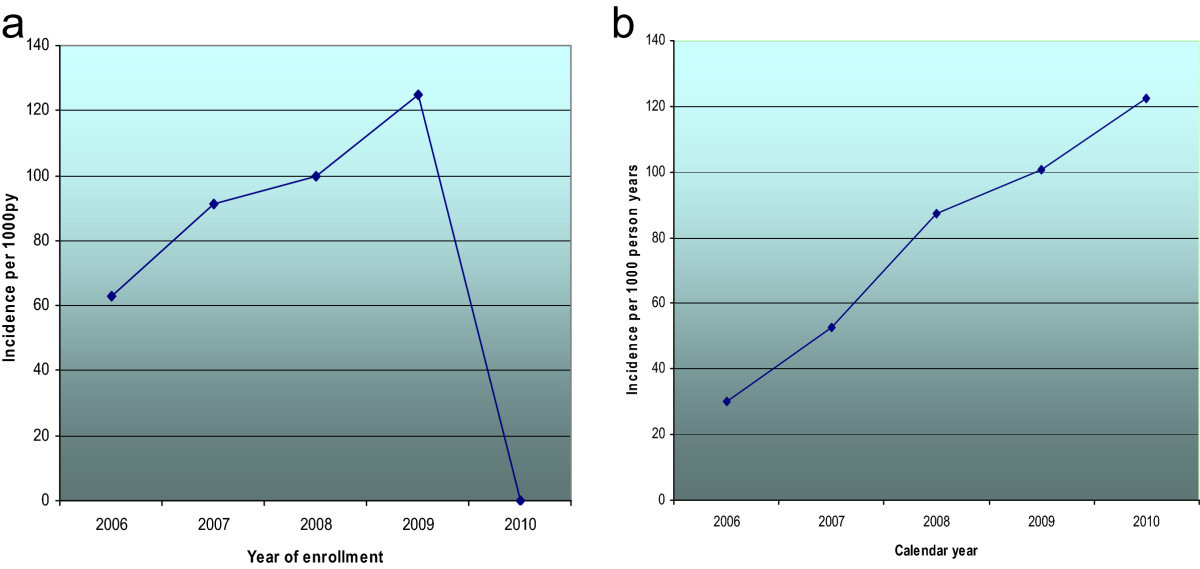
**Incidence of pregnancy by year of enrollment (a) and calendar year (b) among women at Mbarara ISS clinic, western Uganda.**

Women were followed up between June 2006 and January 2010. During this period, total of 36,775 visits for the 3,144 women were entered in the database. In this period, 462 (15%) women experienced a pregnancy event. The mean time to a pregnancy event was 16 months.Seventy five pregnancies occurred among women enrolled in 2006, 175 among women enrolled in 2007, 136 pregnancies in 2008, 76 pregnancies in 2009 and no pregnancies among women enrolled in 2010 (Figure 2a).The overall pregnancy incidence rate was 90.8 pregnancies per 1000 person years (PY) with 95% CI 82.9 – 99.5. The incidence rates per 1000 PY by year of enrollment were 63, 91.3, 100, 125 and 0 for 2006 to 2010 respectively. The incident rates per 1000 PY by calendar year were 29.9, 52.8, 87.4, 100.7 and 122.6 for 2006 to 2010 respectively (Figure 2b).We tested for cohort effects in this clinic population by examining incidence of pregnancy by the year of enrollment, considering each group of women in the same year of enrollment as a cohort. There was no significant trend in incidence of pregnancy (Figure 2a). However, the incidence rate of pregnancy by calendar year increased significantly over time (Figure 2b) and chi square test for trend p value <0.0.001. These annual incidence rates were calculated for all women during the calendar year within which they occurred, regardless of the year of enrollment.

### Predictors of pregnancy

The women aged less than 18 years (HR 10.9 95% CI 3.22-37.15, p < 0.0001) and those aged 18–24 years (HR = 9.93, CI 6.1-16.1, p < 0.0001) were more likely to become pregnant compared to those aged 39 years and above (Table 3). Other factors associated with an increased risk of pregnancy include being single or married, less years of education i.e., having attended primary school education only, unemployed and peasant farmers. Women in a lower income bracket as measured by a monthly income less than 100,000 Uganda shillings (40 USD) or women who did not know their spouse’s HIV serostatus were also more likely to become pregnant compared to the women in the higher income bracket and those who knew their spouse’s HIV serostatus.

**Table 3:** **Rates of pregnancy per 1000 person years with crude hazard ratios for the predictor variables**

Factor	Number of pregnancies	Rates/1000 py	Crude HR (95% CI)	p-value
**Age**				
39 and above	21	16	1	
32 to 38	97	69.5	4.15 (2.52-6.82)	<0.001*
25 to 31	208	132.7	8.23 (5.13-13.19)	<0.001*
18 to 24	133	168	9.93 (6.10-16.12)	<0.001*
Less than 18	3	166	10.9 (3.22-37.15)	<0.001*
**Marital status**				
Widowed/separated	149	60.6	1	
Single	38	110	1.95 (1.34-2.84)	<0.001*
Married	260	125.5	2.09 (1.69-2.69)	<0.001*
**Education**				
Tertiary	18	62	1	
Secondary	84	99	1.66 (0.56- 1.68)	0.008*
Primary	282	102.4	1.65 (1.02-2.66)	0.039*
None	44	60	1.63 (0.87-3.06)	0.129
**Occupation**				
Employed	86	79.6	1	
Unemployed	95	108.5	1.36 (1.02, 1.82)	0.037*
Peasant farming	180	87	1.10 (0.85, 1.43)	0.449
Others	59	99	1.36 (0.89, 1.73)	0.189
**Religion**				
Others	20	88.3	1	
Protestant	219	90	1.02 (0.65-1.61)	0.932
Catholic	128	95.3	1.08 (0.67- 1.72)	0.749
Moslem	45	95	1.07 (0.64-1.82)	0.781
**Income**				
500,000 and above	8	83.8	1	
100,000 – 500,000	20	58.6	0.69 (0.31- 1.58)	0.393
Less than 100,000	263	106.4	1.27 (0.63 - 2.56)	0.506
**WHO disease stage**				
Stage 4	2	24.5	1	
Stage 3	38	73.9	2.91 (0.73-12.1)	0.140
Stage 2	151	94.7	3.74 (0.93-15.1)	0.064
Stage 1	152	82.5	3.25 (0.81-13.1)	0.097
**Spouse status**				
Positive	214	97.2	1	
Negative	15	140.5	1.44 (0.86 -2.44)	0.167
Unknown to woman	71	143.9	1.46 (1.13 – 1.93)	0.004*
**Family planning use**				
No modern FP	339	134.9	1	
Ever used modern FP	70	31.4	0.23 (0.18- 0.30)	<0.001*
**ARV**				
Use	297	90.6	1	
No use	165	91.5	1.01 (0.83-1.22)	0.418

*Significant at 0.05 level.

**Figure 3:**
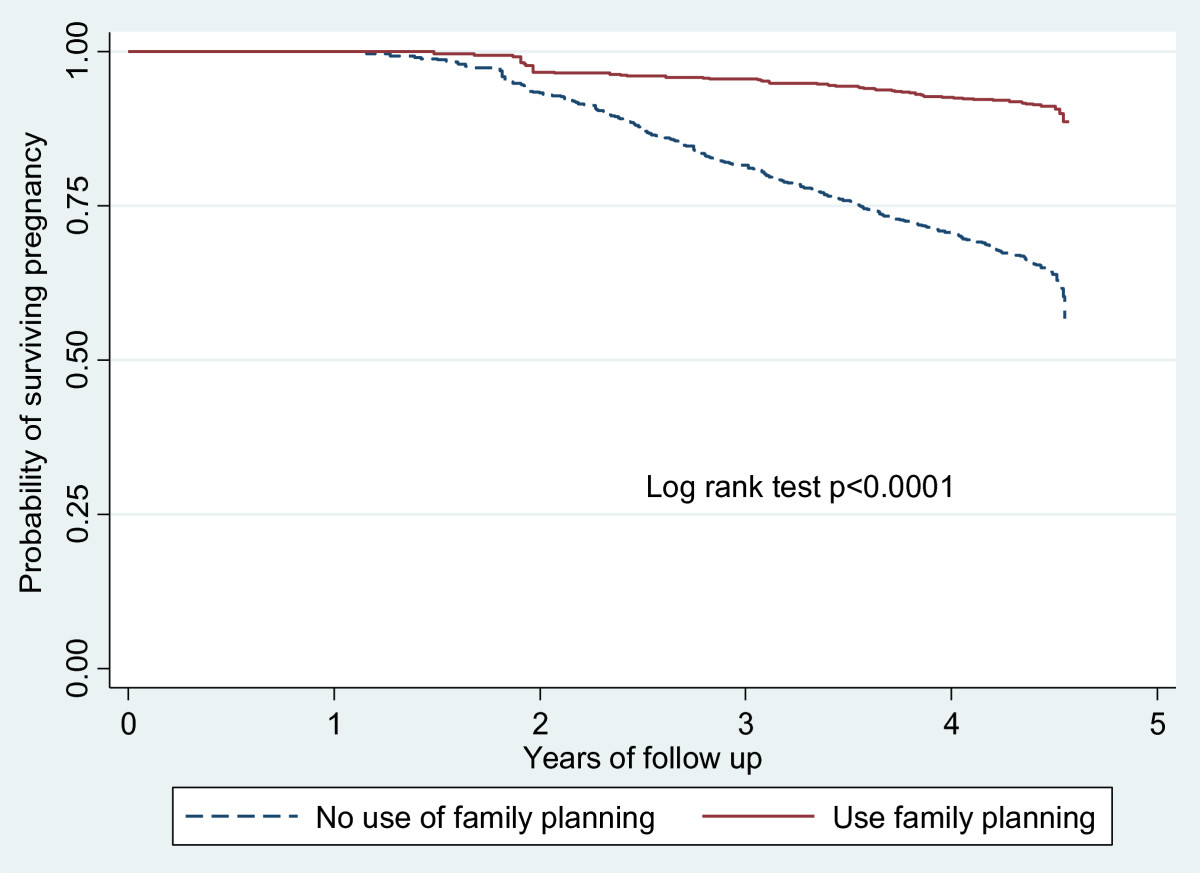
**Kaplan Meier survival curve to show incidence of pregnancy by use of family planning among women of reproductive age attending Mbarara ISS clinic, western Uganda.**

Factors associated with a decreased risk of pregnancy include having ever used any method of family planning (HR = 0.23, CI = 0.18- 0.30, p < 0.0001) and increase of 100 or more in CD4 (CD4 difference) between baseline and most recent CD4 measure. The Kaplan Meier survival curves for women who had ever used and those that had not were significantly different, with a log rank test of p value <0.0001 (Figure 3). The graph shows that by the end of the follow up period, about 80% of the women who had ever used any family planning method were still not pregnant compared to about 60% among those who never used family planning. Women who were married or single were more likely to become pregnant compared to those who were separated or divorced (Table 3). For CD4 count, we compared the baseline measurement with the most recent measure and computed the difference. A CD4 difference of 350 or above was associated with lower incidence of pregnancy with HR 0.34, 95% CI 0.26 – 0.45, p < 0.0001 (Table 4).

**Table 4:** **Rates of pregnancy per 1000 person years with crude Hazard ratios for the predictor variables**

Variable	Pregnancies per year	Rates/1000 PY	Crude HR (95% CI)	p-value
**Calendar year**				
2006	4	29.9	1	
2007	42	52.8	1.77 (0.63 – 4.92)	0.277
2008	127	87.4	2.92 (1.08- 7.91)	0.035*
2009	190	100.7	3.36 (1.25 – 9.07)	0.016*
2010	99	122.6	4.10 (1.5-11.15)	0.006
**Year of enrollment**				
2006	75	63.5	1	
2007	175	91.3	1.43 (1.09 – 1.88	0.009*
2008	136	100	1.57 (1.19 – 2.08)	0.002*
2009	76	125.7	1.97 ( 1.43-2.7)	<0.0001*
2010	0			
**Disclosure**				
No	29	77	1	
Yes	126	102	1.33 (0.89–1.99)	0.160
Missing	307	88.1	1.14 (0.78 – 1.68)	0.485
**Children alive**				
5 and above	274	93.5	1	
2 - 4 children	116	72.7	0.78 (0.63 – 0.97)	0.023*
1 child	72	128.5	1.37 (1.06 - 1.78)	0.016*
**Weight (kg)**				
Less than 50	71	65.1	1	
50 -60	201	97.4	1.49 (1.14-1.96)	0.004*
61 -71	110	93.3	1.43 (1.063-1.93)	0.018*
Above 71	47	72.6	1.11 (0.77 – 1.61)	0.56
**CD4 cell count at enrollment**				
Less than 100	57	77.85	1	
100 – 250	113	97.74	1.21 (0.88-1.66)	0.233
251 – 350	74	98	1.21 (0.86-1.72)	0.265
Greater than 350	185	78	0.97 (0.72-1.35)	0.854
**Difference in CD4 between most recent and enrollment counts**				
Less than 100	58	243.7	1	
100 – 250	8	72.9	0.29 (0.14-0.62)	0.001*
251 - 350	2	45.2	0.18 (0.45- 0.76)	0.019*
Greater than 350	394	84.1	0.34 (0.26 - 0.45)	<0.0001*

*Significant at 0.05 level.

The factors that were not significant predictors of pregnancy include religion, WHO disease stage, ARV use and CD4 cell count at enrollment. The disclosure of HIV status was defined as disclosure to any concerned person and not necessarily their spouse. The results suggest there is a likelihood of a lower incidence of pregnancy among those with more advanced disease compared to those in earlier disease stage but the association was not significant.

We conducted a multiple regression analysis with family planning, age, serostatus of spouse, marital status, education, income and CD4 difference in the model. In the model, use of family planning, younger age, marital status, education and CD4 difference remained significant predictors of pregnancy.

## Discussion

Our study uses routinely collected data to measure incidence and predictors of pregnancy among women of reproductive age attending a large urban HIV clinic serving a predominantly rural population in western Uganda. The data shows incidence of pregnancy among these women has increased consistently for a period of 5 years but is still lower than 195 per 1000 PY in the general population of Ugandan women aged between 35 to 39 years [22]. The increase in incidence of pregnancy has been observed elsewhere in rural Uganda [18, 23] and these studies confirm our findings.

The increasing incidence of pregnancy should be of concern to the clinicians because our data shows at least one third of the women had advanced HIV disease at enrollment in the clinic and may have had advanced disease when they became pregnant. This increases the risk of mother-to-child transmission of HIV and necessitates the early screening and initiation of ART regardless of the CD4 count as currently recommended in the 2013 WHO guidelines for treatment of pregnant and breastfeeding mothers [24] and continuation of ART for life also known as Option B+. Obviously, the implementation of the program of this nature will require significant preparations particularly in high burden countries such as Uganda. There are also likely to be challenges of adherence to treatments among patients who initiate therapy early in the infection and such studies should be conducted to inform policy on treatment recommendations when these programs start.

It is difficult to explain why the incidence of pregnancy is increasing. One of the reasons may be due to ART optimism. Success stories of treatment are now ubiquitous and this may motivate more women to pursue their fertility goals without fear of their health deteriorating. However, this may not be completely true because our data shows no difference in incidence of pregnancy among ART users and non users and a study from rural Uganda has also shown no difference in fertility desires among ART users and non users [25, 26]. Though our data did not show any difference in incidence of pregnancy among ART users and non-users, the evidence supporting ART optimism is from a large ART program implemented in 7 African countries. In this study, incidence of pregnancy among ART users was almost two fold compared to non ART users [27].

Our data may not fully explain the increasing incidence of pregnancy but clearly we are now faced with the increasing need to integrate reproductive health into HIV care programs. Care givers will need to assess their clients’ fertility intentions and incorporate reproductive counseling into the routine care to cater for their reproductive needs especially as HIV care programs mature and patients. For instance, less than 40% of the clients had ever used a family planning method.

Studies also suggest that a majority of pregnancies in rural Africa are unintended and many HIV infected women perceive themselves as infertile [28]. This lack of awareness about fertility potential may be responsible for the large majority of the unintended pregnancies and hence the lack of difference in incidence of pregnancy among women receiving ART compared to those not receiving. Also, a recent clinical trial of pre-exposure prophylaxis among HIV uninfected women in sero-discordant partnerships also showed no significant difference in incidence of pregnancy among women in the placebo and treatment arms [29].

The factors that were associated with incidence of pregnancy in our cohort were young age, marital status, lack of knowledge of spouse HIV serostatus, lower socio economic status and use of family planning. These factors are similar to those that predict pregnancy in the general population. For instance, younger women were more likely to get pregnant compared to the older women. This observation has also been made in previous studies in Africa [18, 27, 30]. These observations are expected because women are generally most fertile between the ages of 20 and 24 years and as they get older the likelihood of getting pregnant declines. It is also likely that younger age is associated with more fertility desire. Among the youth in urban United States, HIV serostatus did not diminish the fertility desires of the female youth [31] but studies of this nature have not been conducted in Africa.

Widowed or separated women were less likely to become pregnant compared to those who were single or married. This finding is also consistent with earlier studies in Africa that have shown that widowed or divorced women had difficulty remarrying because they were afraid of infecting their new partners [28, 32]. Women who did not know their spouse’s HIV serostatus were more likely to become pregnant compared to those who knew. Also, disclosure was not related to incidence of pregnancy. The finding contradicts other studies that show disclosure is associated with higher incidence of pregnancy [23]. Instead, lack of knowledge of spouse HIV status was associated with higher incidence of pregnancy. A significant proportion of the women in our data set had missing disclosure status and this may have contributed to the null findings. However, disclosure has been associated with positive outcomes such as participation in PMTCT programs [33] and therefore should be encouraged using couple counseling approaches.

We were not able to measure fertility desire in this study since our data is obtained from a database designed to track patients in a routine care and treatment program. Therefore, we are not able to establish whether the higher likelihood of pregnancy among women of a lower socio economic status is attached to a higher fertility desire, or whether these pregnancies are mostly unwanted. Future studies should address this question.

Women who have fewer children were more likely to become pregnant compared to women who had more. Prior studies in sub Saharan Africa have shown that reported death of a child or a history of spontaneous abortion were associated with a higher desire to get pregnant [26, 34, 35], however our study was not able to collect these variables as predictors. It is not clear what the impact of number of HIV positive children would have as our data on HIV positive children was not reliable for use in this analysis. We would expect that mothers who have HIV positive children may be motivated to want to have another child with the hope the child will be negative.

Larger increases in CD4 count were associated with lower probability of pregnancy. The observation could not be due to reverse causality as pregnancy itself does not contribute to HIV disease progression [20, 36–38]. Women who had ever used any modern family planning method during the follow up period were less likely to become pregnant. The prevalence of contraceptive use was however very low at 37%. Our data did not capture the specific types of family planning such as condoms or hormonal contraceptive methods and hence we are not able to establish the predominant methods being used at the clinic. However a study done at this clinic showed higher prevalence of contraceptive use particularly among women receiving ART compared to those not on treatment and also more likely to use the barrier methods of contraception [39].

Our study has shed light on the increasing incidence of pregnancy at a treatment center at a large urban clinic in Uganda, but it has several weaknesses. First, we dropped 31% of the data because the mothers’ pregnancy records were missing from the data set. However, we have no reason to believe this could have biased the analyses significantly since the demographics of these mothers with missing records were similar to those retained in the analysis. Second, we did not collect data on fertility intentions and also lacked specific data on use of the types of contraceptive methods vis-à-vis barrier and hormonal methods. Thirdly pregnancy was not always confirmed with urine HCG but was determined by history and clinical examination on subsequent visits and also, it was not possible to establish the exact dates when pregnancy was first noted from the data set, especially given that our patients do not visit the clinic at even time intervals. Lastly, our analysis does not address pregnancy outcomes and or the subsequent pregnancies that occurred during the follow up period. Future studies should be conducted to explore these issues.

The strength of this paper is that we are able to efficiently use routinely collected data to demonstrate trends in incidence of pregnancy among women receiving ART in at a large clinic. Our work is among the few papers that have demonstrated an increase in incidence of pregnancy.

In conclusion, caregivers should integrate reproductive health services should into HIV clinics to cater for the needs of these women. The reproductive health counseling will likely need to involve the spouses as well. Also, carefully designed prospective studies should be conducted to show the relation between fertility intentions and practice related to the first pregnancy after ART has started but also for subsequent or repeat pregnancies.

## Authors’ original submitted files for images

Below are the links to the authors’ original submitted files for images. Authors’ original file for figure 1 Authors’ original file for figure 2 Authors’ original file for figure 3
