# Supplementary figures and images for: Increasing incidence of pregnancy among women receiving HIV care and treatment at a large urban facility in western Uganda
Source: Reprod Health. 2014 Dec 6;11:81. doi: 10.1186/1742-4755-11-81 (PMC4364564; doi:10.1186/1742-4755-11-81)

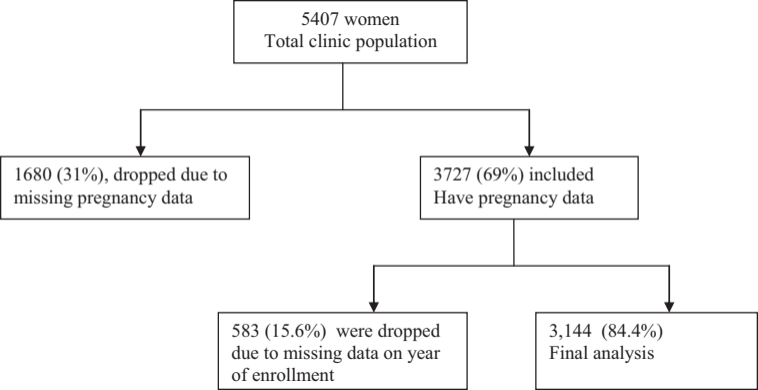

Supplement: Supplementary file 1 — Authors’ original file for figure 1 [file 12978_2012_342_MOESM1_ESM.pdf]

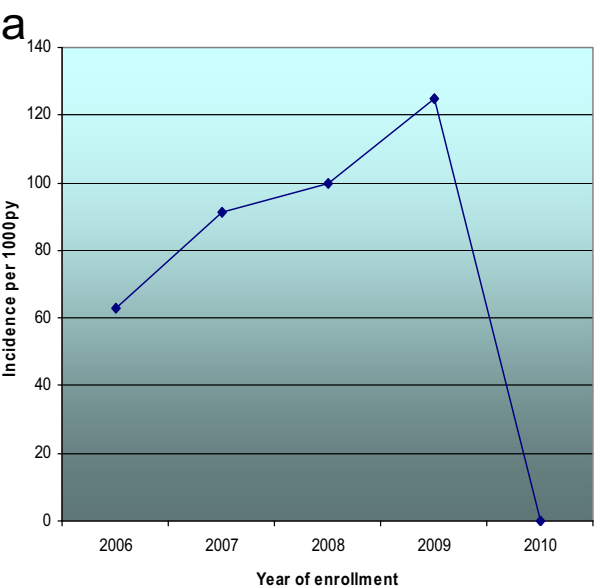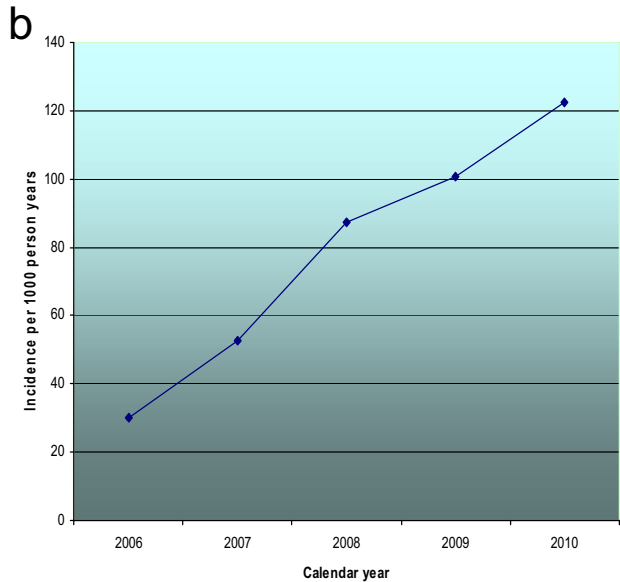

Supplement: Supplementary file 2 — Authors’ original file for figure 2 [file 12978_2012_342_MOESM2_ESM.pdf]

Probability of surviving pregnancy

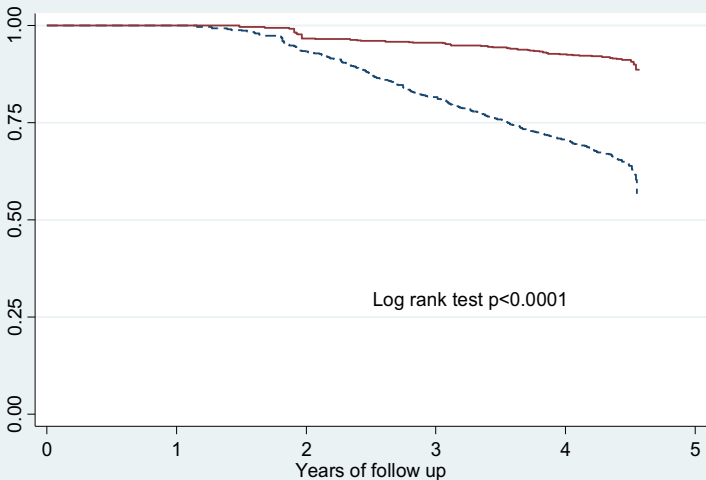

--- No use of family planning

— Use family planning

Supplement: Supplementary file 3 — Authors’ original file for figure 3 [file 12978_2012_342_MOESM3_ESM.pdf]
